# Adiaspore development and morphological characteristics in a mouse adiaspiromycosis model

**DOI:** 10.1186/s13567-020-00844-3

**Published:** 2020-09-15

**Authors:** Asuka Takeshige, Mie Nakano, Daisuke Kondoh, Yuma Tanaka, Akio Sekiya, Takashi Yaguchi, Hidefumi Furuoka, Takahito Toyotome

**Affiliations:** 1 Department of Veterinary Medicine, Obihiro University of Agriculture and Veterinary Medicine, Inada-cho, Obihiro, Hokkaido 080-8555 Japan; 2 Medical Mycology Research Center, Chiba University, 1-8-1 Inohana, Chuo-ku, Chiba, 260-8673 Japan; 3 Diagnostic Center for Animal Health and Food Safety, Obihiro University of Agriculture and Veterinary Medicine, Inada-cho, Obihiro, Hokkaido 080-8555 Japan

**Keywords:** adiaspiromycosis, adiaspore, *Emmonsia crescens*

## Abstract

Lesions of adiaspiromycosis, a respiratory disease affecting wild animals, have been found mainly in dead mammals and free-living mammals captured for surveillance. No report has described an investigation of adiaspore formation progress in the lung. After establishing an experimental mouse model of intratracheal adiaspiromycosis infection with the causative agent *Emmonsia crescens*, we observed adiaspore development. The spores grew and reached a plateau of growth at 70 days post-infection. The median adiaspore diameter showed a plateau of around 40 μm. The characteristic three-layer cell-wall structure of adiaspores was observed in the lung at 70 days post-infection. We examined infection with a few spores, which revealed that adiaspores in the mouse lung progressed from intratracheal infection of at least 400 spores. Moreover, we developed adiaspores in vitro by culture in fetal bovine serum. Although most spores broke, some large spores were intact. They reached about 50 μm diameter. Thick cell walls and dense granules were found as common points between in vitro adiaspores and in vivo adiaspores. These models are expected to be useful for additional investigations of *E. crescens* adiaspores and adiaspiromycosis.

## Introduction

*Emmonsia crescens* (*Ajellomyces crescens*), a dimorphic fungus found in soil worldwide [1], is known to be the causative agent of adiaspiromycosis, a pulmonary disease causing granulomatous lesions in humans [2] and animals, especially in small mammals [1–7] and rarely in large animals [8, 9]. Although the prevalence of adiaspiromycosis in animals remains unknown, Borman reported adiaspiromycosis in 28.7% of free-living wild mammals from the southwestern UK [10]. Reported human cases of adiaspiromycosis are few, with disease courses varying from mild to fatal [2]. Studies have been done of wild animals captured with traps or found dead incidentally, then subsequently diagnosed as having adiaspiromycosis during necropsy. Because no report of the literature has described a study of the disease course from spore acquisition to mature adiaspore formation in the lung, related details have remained unknown.

At around 25 °C, *E. crescens* grows as hyphae. However, the fungus forms adiaspores in mammalian hosts at around 37 °C [11]. A few infection models have been reported. An inhalation model has been used with rabbits [12] to analyze humoral immunological responses. Experimental mouse models of adiaspiromycosis using intraperitoneal infection routes have been described in the literature [4, 13, 14]. The models used neither an intranasal nor an intratracheal route. Adiaspore enlargement dynamics have been examined in mice using an intraperitoneal model, but those circumstances differ markedly from those in the lung. For the present study, after we established an experimental mouse model of adiaspiromycosis via the intratracheal route, we examined adiaspore development in vivo. We also used different inoculation numbers of *E. crescens* spores from 400 to 4 × 10^4^ to assess their pathogenicity. We examined the adiaspore morphology using optical and electron microscopy. Furthermore, we found new adiaspore development procedures in vitro and compared associated morphological characteristics of the adiaspores to those of adiaspores formed in vivo.

## Materials and methods

### Fungal strains and the growth condition

Spores of *Emmonsia crescens* IFM 46988 (CBS475.77, UAMG127) used for this study were provided by the Medical Mycology Research Center, Chiba University. The strain was cultured routinely on potato dextrose agar slants at 25 °C for around 4 weeks. A spore suspension was prepared as described earlier [15].

### Intratracheal infection of mice with *E. crescens* and tissue section preparation

All animal experiments were approved by the Animal Care and Use Committee of Obihiro University of Agriculture and Veterinary Medicine (Nos. 27-152, 28-22, 29-170). The intratracheal intubation procedure was described in an earlier report [16]. We used C57BL/6N mice (male, 7 weeks) supplied by Charles River Laboratories Japan, Inc. After confirming correct intubation, 25 μL of spore suspension in saline was introduced through a catheter. We used a larger burden (4 × 10^6^ spores/mouse) and smaller burdens (4 × 10^2^, 4 × 10^3^, 4 × 10^4^ spores/mouse) as spore numbers for intratracheal infection. After infection with the larger burden, mice were killed at different time points (three mice each at 1, 2, 3, 4, 6, 8, 10, 30, 50, and 70 days post-infection (dpi) and one mouse each at 100 and 150 dpi). After tissues including those of the lung, heart, liver, spleen, and kidney were collected, they were fixed with 10% formalin and were subsequently embedded in paraffin. Paraffin blocks were sliced (approximately 3 μm thick) and processed using usual deparaffinization and hydration procedures. Each section was stained with hematoxylin and eosin (HE) or periodic acid–Schiff (PAS) procedure. For small burden infections, three mice each were infected and later killed at 70 dpi. Body weight was measured twice a week after infection. For comprehensive analyses to detect infection foci with smaller burden sizes, six section samples were prepared for every 50–80 μm thickness of lung paraffin blocks and were examined using HE staining.

### TEM analysis

Lungs of mice inoculated 4 × 10^6^ spores were used for transmission electron microscopy (TEM) analysis. The lungs were collected at 30, 50, and 70 dpi, fixed with 2.4% glutaraldehyde (pH 7.4), immersed in 1% phosphate-buffered osmium tetroxide (pH 7.4), dehydrated, substituted with propylene oxide, and were subsequently embedded in epoxy resin. After ultrathin sections (approximately 90 nm thick) were cut using a diamond knife and ultramicrotome, they were observed using a transmission electron microscope (HT7700; Hitachi High Technologies Corp., Tokyo, Japan) without uranyl acetate or lead citrate staining.

### In vitro adiaspore induction

After 1 × 10^5^ spores were inoculated into 2 mL of fetal bovine serum (FBS) in a 35-mm dish, they were cultured at 37 °C for 60 days for TEM analysis or 71 days under 5% CO_2_ condition for determination of the adiaspore diameters. New 2 mL FBS was added every 30 days. To ascertain the adiaspore diameters, images were taken at 8, 30, 50, and 71 days after inoculation using a fluorescence microscope (BZ-9000; Keyence Co., Osaka, Japan). For TEM analysis, spores were fixed at 60 days after inoculation with 4% paraformaldehyde in phosphate-buffered saline (Fujifilm Wako Pure Chemical Corp., Osaka, Japan) followed by 2.4% glutaraldehyde. Then they were immersed in 1% phosphate-buffered osmium tetroxide (pH 7.4). The precipitated spores were dehydrated, embedded in LR White resin, and then cut into semi-thin sections (approximately 1 μm thick) for light microscopic observation with toluidine blue staining, or into ultrathin sections for analysis using a transmission electron microscope (HT7700; Hitachi High Technologies Corp.).

## Results

### Adiaspores developed in the lung examined using an experimental mouse model of intratracheal infection

**Figure 1:**
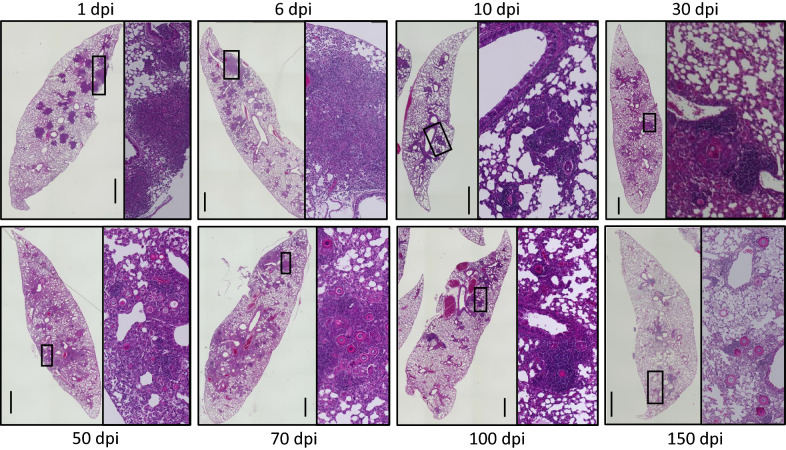
**Mouse lung lobes infected with** ***E. crescens***. Fields of squares are shown in the right panel in each image. Scale bar = 1 mm.

**Figure 2:**
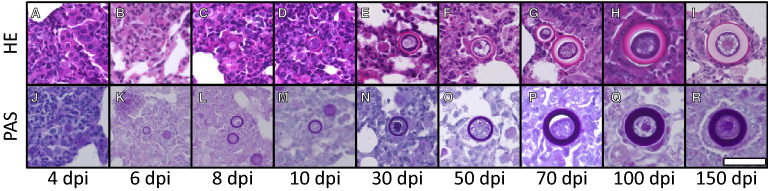
**Adiaspore development in mouse lung.** Specimens were stained with HE (**A**–**I**) or PAS procedure (**J**–**R**). Scale bar = 50 µm. All images are of the same scale.

After infection with a higher burden (4 × 10^6^ spores/mouse), mice showed no symptoms. Their body weights increased gradually along with those of uninfected mice (Additional file 1). The *E. crescens* spores and adiaspores associated with cellular infiltration were found in the lung (Figure 1), but not in the heart, liver, spleen, or kidney. The spores increased in size and showed structures similar to those of adiaspores (Figure 2), suggesting that the model induced adiaspores in mice by instillation through the intratracheal route.

### Maturation of adiaspores from spores in vivo

**Figure 3:**
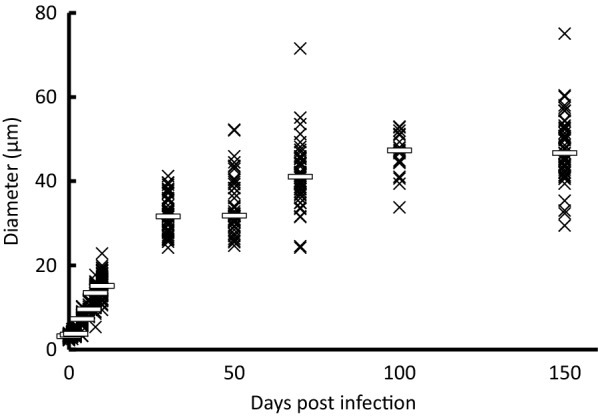
**Spore and adiaspore diameters observed in mouse lung.** White bars show the median at each time point.

**Figure 4:**
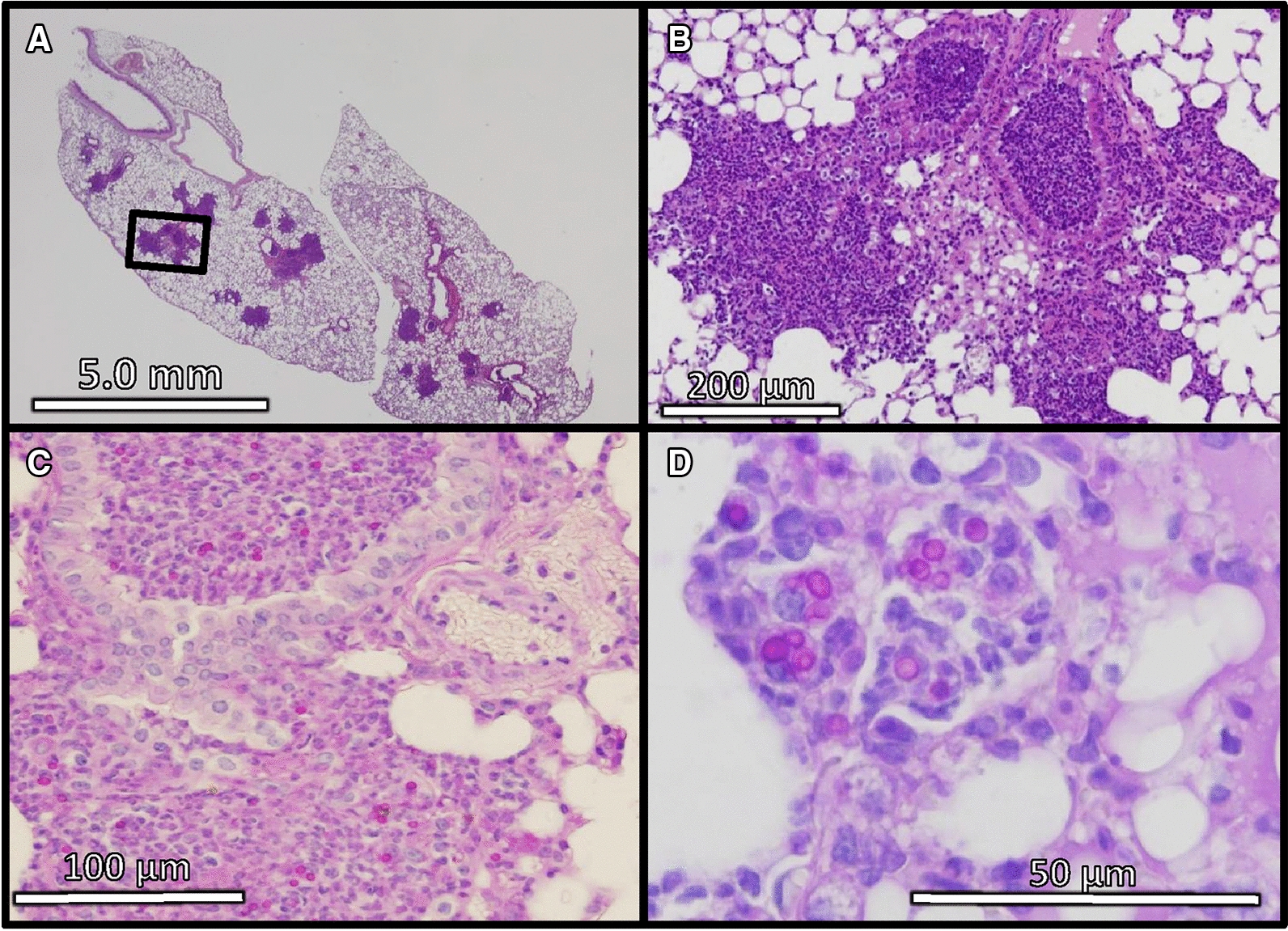
**Infection foci in mouse lung at 1** **day after** ***E. crescens*** **infection.** These specimens were stained with HE (**A**, **B**) and PAS procedure (**C**, **D**). The field of a square in **A** is magnified in **B**.

Spore diameters in the lung were measured at each time point (Figure 3). At 1 dpi, inflammation mainly from accumulated neutrophils was observed in the infected lungs (Figure 4A, B). Inoculated spores were found in and around the bronchi and the alveoli (Figure 4C). Some spores were phagocytosed by macrophages (Figure 4D). The median spore sizes at 1 dpi (3.6 μm) was similar to that of recovered spores from potato dextrose agar slant in vitro (3.2 μm mean diameter). Enlarged spores were found in the lung at 4 dpi (median 7.2 μm, Figure 3). The thick cell walls of some spores were observed clearly, as portrayed in Figure 2A, J. Infiltration of lymphocytes around bronchi and blood vessels were observed mainly at 8 dpi. Some spores were surrounded by multinucleated giant cells (arrows in Additional file 2). The median spore size reached 13.5 μm at 8 dpi (Figure 3). The average spore size reached 41.1 ± 7.5 μm at 70 dpi. The average size did not exceed 50 μm until 150 dpi in the strain (Figure 3).

**Figure 5:**
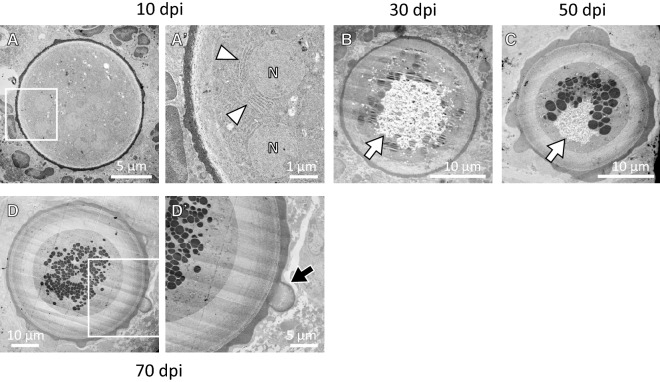
**TEM images of adiaspores in mouse lung at 10 (A, A′), 30 (B), 50 (C), and 70 (D, D′) days post-infection.** Fields surrounded with a white square in **A**–**D** are magnified respectively in **A**′–**D**′. White arrowheads in **A**′ indicate the endoplasmic reticulum-like structure surrounding the nucleus-like structure (N in **A**′). White arrows in **B** and **C** and a black arrow in **D**′ respectively point to unstructured materials and a bleb-like structure.

The three-layered cell wall structure was observed clearly in most adiaspores at 70 dpi (Figure 2G). At 100 and 150 dpi, some adiaspores showed a multilayered cell wall structure (Figure 2H, I). The outermost layer of the cell wall showed a bleb-like structure on some adiaspores (Figure 2F, G). As early as 4 dpi, an internal space with unstructured materials was observed in some adiaspores (Additional file 3). The area expanded gradually during infection. In contrast to HE images, granular materials in the space were strongly stained with PAS stain (Additional file 4). At 30 dpi and later, some adiaspores were found with debris-like materials or were empty (Additional file 5). Some showed that the cell wall structure had partly ruptured (left image in Additional file 5). We conducted TEM analysis for mouse lungs recovered at 10, 30, 50, and 70 dpi. Adiaspores observed at 10 dpi retained intracellular contents and a membrane structure of organelles (Figure 5A, A′), which are similar to the endoplasmic reticula surrounding the nucleus. Although several nucleus-like structures were observed in an adiaspore, cellular membranes to septate these nuclei were not found in the adiaspore (Figure 5A, A′). Similarly to the results observed under an optical microscope, adiaspores showed internal space with unstructured materials (arrows in Figure 5B, C). A bleb-like structure on the outermost layer of the cell wall and a three-layered cell wall structure were confirmed using ultrastructural analysis (Figure 5D′). Most adiaspores were found to have readily visible electron-dense granules at 30 dpi and higher resolution (Figure 5B–D). We identified characteristics of *E. crescens* development from spore to adiaspore using this mouse model with infection via the intratracheal route.

### Adiaspores induced by fetal bovine serum in vitro

**Figure 6:**
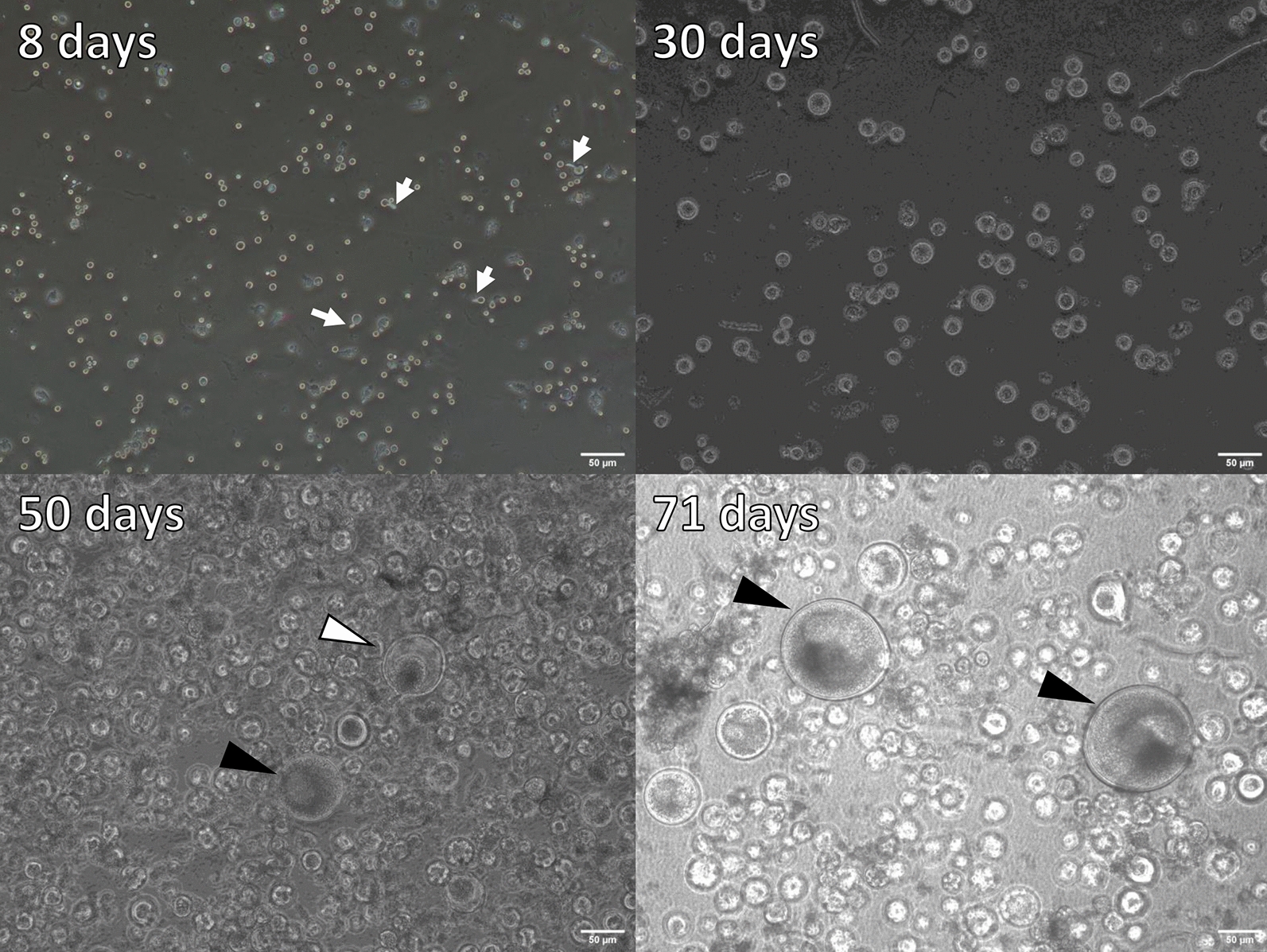
**Adiaspore development in FBS in vitro.** Culture durations were shown in each image. White arrows indicate germtubes. Arrowheads indicate remarkably large adiaspores. Scale bar = 50 µm.

**Figure 7:**
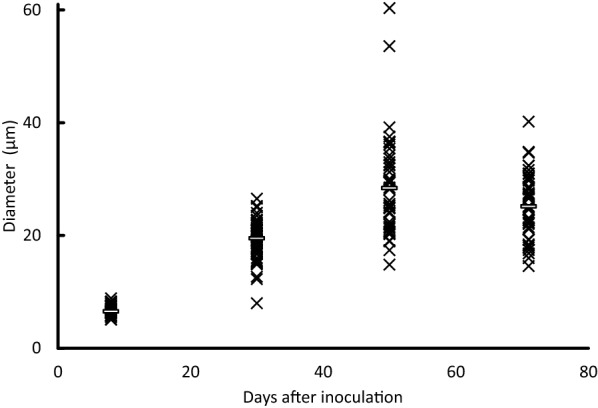
**Diameters of adiaspores cultured in vitro.** White bars show the median at each time point.

**Figure 8:**
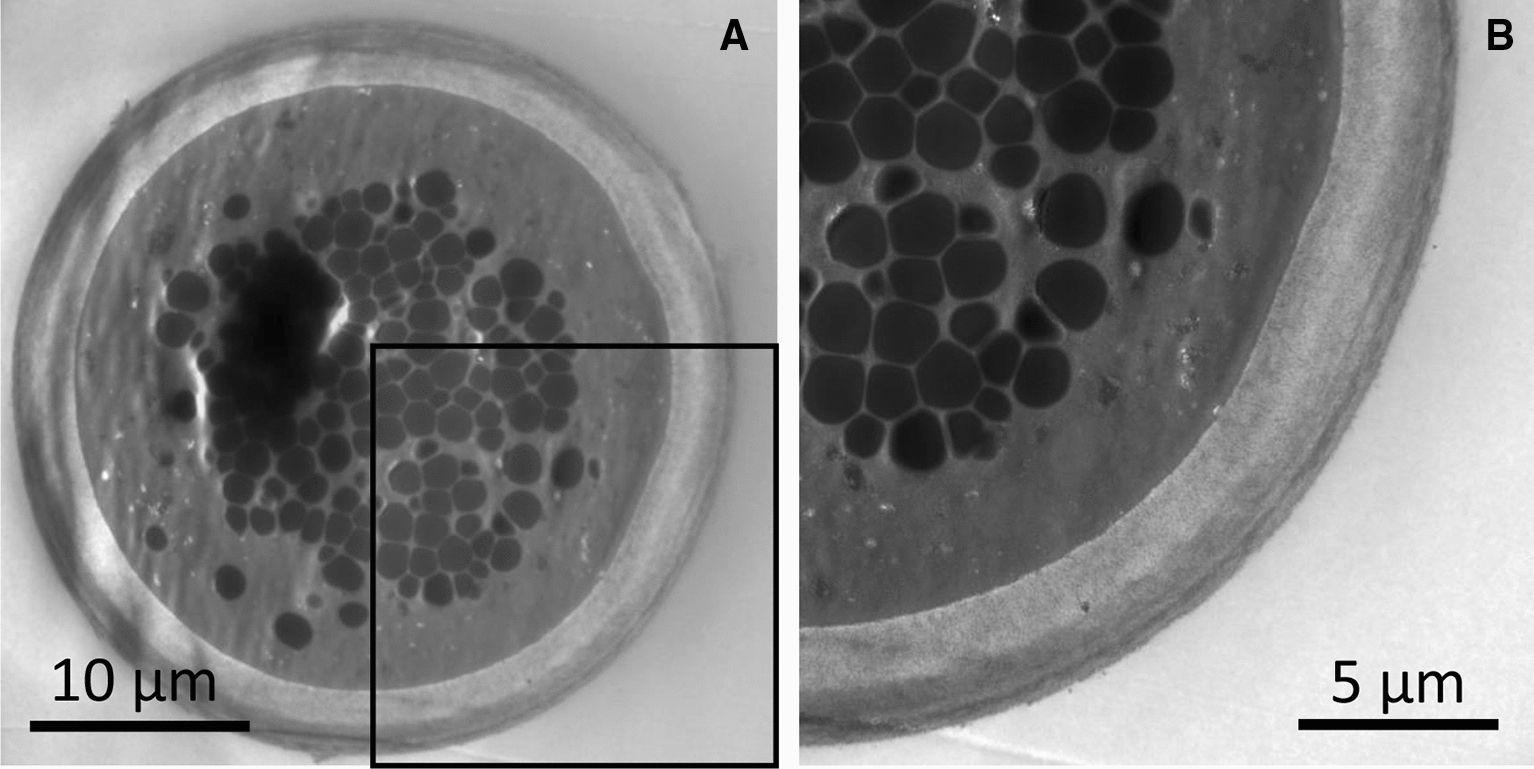
**Adiaspores cultured in FBS for 60** **days in vitro.** The magnified image in a black frame of **A** is shown in **B**.

We examined adiaspore formation in vitro, observed at 8, 30, 50, and 71 days post-inoculation (Figures 6 and 7). As the culturing time increased, the spores became larger. They reached a plateau in terms of size at 50 days post-inoculation (Figure 7). At 8 days post-inoculation, some spores formed a germ tube-like protrusion (white arrows in Figure 6). At 50 days and 71 days post-inoculation, although most of the adiaspores were of ca. 30 µm in diameter, some adiaspores had grown remarkably large under the culture (arrowheads in Figure 6). Among our observations, the largest adiaspore at 71 days post-inoculation had diameter of greater than 120 µm in. The cell wall indicated by a white arrow in Figure 6 was ruptured, thereby resembling the cell shown in the left image of Additional file 5. Adiaspores cultured for 60 days are shown in Figure 8 (TEM analysis) and in Additional file 6 (stained with toluidine blue). As shown in Figure 6, some adiaspores induced in vitro showed a three-layer structure of the cell wall and electron-dense granules in the spores. They exhibited similar structural features to those of adiaspores formed in the lung on the experimental mouse model.

### Infection focus formation with small burdens of *E. crescens* spores in mouse lung

Next, we examined *E. crescens* infections with a small burden size (4 × 10^2^, 4 × 10^3^, or 4 × 10^4^ spores/mouse) through the intratracheal route to elucidate how a few spores establish infection in mouse lung tissues. Body weights increased as they did for an uninfected mouse (Additional file 7); no symptom was apparent. We used three mice for each inoculation burden and observed the infection foci in mouse lungs at 70 dpi after infection. Adiaspores were found in the lung of each mouse that had been infected with 4 × 10^2^ and 4 × 10^3^ spores. Under the condition of infection with 4 × 10^4^ spores, although adiaspores were not found in every mouse, the infiltration of lymphocytes (10 foci) was found. These results suggest that *E. crescens* can form infection foci and adiaspores in mouse lung under a small burden: even at 400 spores/mouse.

## Discussion

For this study, we established an experimental mouse model for intratracheal infection with *E. crescens*. Subsequently, we observed adiaspore development in mice lungs. Adiaspores in mice were observed in the lung only, which is consistent with cases of wild animals reported earlier [5, 6, 8].

Additionally, we established a novel in vitro culturing model for adiaspore formation. The model uses FBS as the medium. Adiaspores were maintained for more than 70 days, until 150 days in the model (data not shown). As described earlier, solid media such as brain–heart infusion agar have been used frequently for in vitro culture [10, 11]. Solid media present long-term culture difficulty because of drying. For a model using a liquid medium (FBS), medium addition or culture medium replacement can be done for long-term culturing. Moreover, the collection of adiaspore from FBS is easier than collection from solid media. Therefore, in vitro culture is useful for additional analyses of adiaspores, such as gene expression analysis. For the present study, we compared the adiaspore morphologies of those grown through infection of the lung and those grown through in vitro culture. Both models are complementary, but not perfect: in vitro culture model entails no host immunological response; in vivo infection presents difficulty when analyzing the reactions and changes of *E. crescens*. Parallel analyses using both models are expected to be beneficial for additional understanding of *E. crescens* and the diseases they cause.

It is particularly interesting that *E. crescens* infection with a small burden size, even as few as 400 spores, formed an infection focus. Although *E. crescens* is not dispersed in the bloodstream or other organs, the species can form infection foci with small burdens in immunocompetent hosts. In a natural environment with *E. crescens*, small mammals including rodents are incidentally exposed to *E. crescens* spores. The inhaled spores form adiaspores in their lung.

Infected mice in the model showed no symptoms, which is consistent with most reported cases, showing a lack of readily apparent symptoms from infection. Schaffer-White and colleagues reported two cases of Northern hairy-nosed wombats (*Lasiorhinus krefftii*) [17], and described that the cause of death of one wombat was pulmonary adiaspiromycosis. Northern hairy-nosed wombats might be less resistant to the causative agents. Susceptibility to *E. crescens* and infection severity are thought to vary among animals, suggesting that further investigations should be conducted to ascertain the pathogenicity to wild animals, including endangered animals.

Our experimental model induces adiaspores with average diameter of around 50 μm. Adiaspores of *E. crescens* in wild animal cases are around 300 μm (50–500 μm) diameter [18]. The reason for the small maximum adiaspore size in our model remains unknown. The body or lung size of the host might limit the adiaspore size, but the mean diameter of adiaspores was found to be greater than 300 μm in small mammals such as field voles (*Microtus agrestis*) and bank voles (*Clethrionomys glareolus*) [19]. Additional study using multiple strains is warranted because only one strain was used for this study.

Most adiaspores described in earlier reports were empty or with residual eosinophilic components [6, 7, 20, 21]. In contrast to them, most adiaspores observed in our study remained filled with basophilic components. Empty adiaspores and adiaspores with residual eosinophilic components were observed more frequently in the lungs at 150 dpi compared with other earlier time points, suggesting that most cases are found in wild animals after lengthy infection.

Hughes and Borman reported adiaspiromycosis in a wild European rabbit (*Oryctolagus cuniculus*), which showed a bleb-like structure of the outermost layer on the surface. Bleb-like structures similar to those were found during our observations [22]. Multinucleate giant cells surrounded adiaspores shown in Additional file 2 resemble those of an earlier report of a study conducted by Hughes and Borman [22].

We were able to detect membrane structures of organelles in adiaspores at 10 dpi. At 30 dpi and higher resolution, instead of membrane structures, electron-dense granules were observed in the center of adiaspores. Those lipofuscin-like structures were also observed in adiaspores that had been formed in vitro. Similar structures were found in adiaspores in a wild Franklin’s ground squirrel [23]. Electron microscopic observation of the granules revealed similar characteristics to those found for lipofuscin: dense particles observed as a brown-yellow material under optical microscopy (Additional file 6). Lipofuscin is well known to be an age-related material. It is composed mainly of lipids and proteins that are highly oxidized. These data suggest that adiaspore aging produces a lipofuscin-like structure in vivo and in vitro.

**Figure 9:**
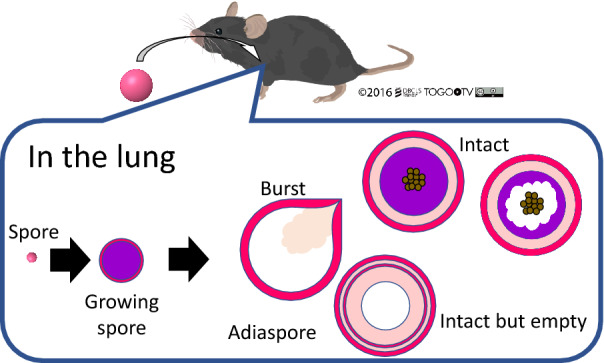
**Graphical summary of adiaspore development in vivo.**

Results and a model of the adiaspore fate from the time-course analysis in vivo are presented in Figure 9. Animals acquire *E. crescens* spores through inhalation. A few spores form infection foci. After infection, spores expand in size and form adiaspore structures with a three-layer cell wall including an outermost bleb-like layer. During and after formation, some adiaspore cell walls rupture, thereby releasing the spore contents. Empty adiaspores are only found in the lung of animals with adiaspiromycosis after long-term infection.

## Supplementary information

**Additional file 1. Body weights of mice infected with 4** **×** **10**^**6**^ **spores or uninfected.** Error bars represent standard deviations of the means.

**Additional file 2. Adiaspores surrounded by multinucleate giant cells at 8 dpi.** Arrows and arrowheads respectively indicate multinucleate giant cells and surrounded adiaspores.

**Additional file 3. Adiaspores in mouse lungs at 4, 10, 30, and 50** **dpi.**

**Additional file 4. Representative adiaspores in which granular materials (arrow) were observed.** The adiaspores are those of mouse lungs at 70 dpi, stained using PAS procedure.

**Additional file 5. Adiaspores at 30 dpi (left) and 70 dpi (right) in mouse lungs.** The adiaspore found at 30 dpi was not intact, but it retained debris-like materials. The adiaspore (arrow) found at 70 dpi was empty.

**Additional file 6. Adiaspore development in FBS in vitro.** Adiaspores were stained with toluidine blue.

**Additional file 7. Body weights of mice infected with 4** **×** **10**^**2**^**, 4** **×** **10**^**3**^**, or 4** **×** **10**^**4**^ **spores, or uninfected.** The error bars represent standard deviations of the means.

## Data Availability

Data supporting the conclusions of this article are included within the article and its additional files.

## Abbreviations

dpi: days post infection
FBS: fetal bovine serum
HE: hematoxylin and eosin
PAS: periodic acid–Schiff
TEM: transmission electron microscopy

## References

[1] Emmons CW, Jellison WL (1960). *Emmonsia crescens* sp. n. and adiaspiromycosis (haplomycosis) in mammals. Ann NY Acad Sci.

[2] Anstead GM, Sutton DA, Graybill JR (2012). Adiaspiromycosis causing respiratory failure and a review of human infections due to *Emmonsia* and *Chrysosporium* spp. J Clin Microbiol.

[3] Bakerspigel A (1961). Haplomycosis (Adiaspiromycosis) in *Sorex*. Can J Microbiol.

[4] Otcenásek M, Dvorák J (1967). The effect of Fungizone on experimental adiaspiromycosis of laboratory mice. Mycopathol Mycol Appl.

[5] Taniyama H, Furuoka H, Matsui T, Ono T (1985). Two cases of adiaspiromycosis in the Japanese pika (*Ochotona hyperborea yesoensis* Kishida). Jpn J Vet Sci.

[6] Nakano M, Yamaguchi E, Kimoto M (2017). Pathological study of adiaspiromycosis in Eurasian red squirrel (*Sciurus vulgaris orientis*) and brown rat (*Rattus norvegicus*) caught in Tokachi district, Hokkaido. Jpn J Zoo Wildl Med.

[7] Simpson VR, Davison NJ, Dagleish MP (2019). Causes of mortality and lesions observed post mortem in European moles (*Talpa europaea*) in Cornwall, south-west England. J Comp Pathol.

[8] Matsuda K, Niki H, Yukawa A (2015). First detection of adiaspiromycosis in the lungs of a deer. J Vet Med Sci.

[9] Pusterla N, Pesavento PA, Leutenegger CM (2002). Disseminated pulmonary adiaspiromycosis caused by *Emmonsia crescens* in a horse. Equine Vet J.

[10] Borman AM, Simpson VR, Palmer MD, Linton CJ, Johnson EM (2009). Adiaspiromycosis due to *Emmonsia crescens* is widespread in native British mammals. Mycopathologia.

[11] Zlatanov ZL, Genov T (1975). Isolation of *Emmonsia crescens* Emmons et Jellison 1960 from small mammals in Bulgaria. Mycopathologia.

[12] Cano RJ, Taylor JJ (1974). Experimental adiaspiromycosis in rabbits: host response to *Chrysosporium parvum* and *C. parvum* var. *crescens* antigens. Sabouraudia.

[13] Hejtmánek M (1976). Scanning electron microscopy of experimental adiaspiromycosis. Mycopathologia.

[14] Slais J (1976). Histopathological changes and the genesis of adiaspiromycomas in mice infected intraperitoneally with *Emmonsia crescens* Emmons et Jellison, 1960. Folia Parasitol.

[15] Toyotome T, Adachi Y, Watanabe A (2008). Activator protein 1 is triggered by *Aspergillus fumigatus* beta-glucans surface-exposed during specific growth stages. Microb Pathog.

[16] Watanabe A, Hashimoto Y, Ochiai E (2009). A simple method for confirming correct endotracheal intubation in mice. Lab Anim.

[17] Schaffer-White AB, Harper D, Mayhew M (2017). Pulmonary adiaspiromycosis in critically endangered northern hairy-nosed wombats (*Lasiorhinus krefftii*). Aust Vet J.

[18] Sigler L, Hay RJ, Merz WG (2005). Adiaspiromycosis and other infections caused by *Emmonsia* species. Topley and Wilson’s microbiology and microbial infections.

[19] Hubálek Z (1999). Emmonsiosis of wild rodents and insectivores in Czechland. J Wildl Dis.

[20] Simpson VR, Tomlinson AJ, Stevenson K (2016). A post-mortem study of respiratory disease in small mustelids in south-west England. BMC Vet Res.

[21] Malatesta D, Simpson VR, Fontanesi L (2014). First description of adiaspiromycosis in an Eurasian otter (*Lutra lutra*) in Italy. Vet Ital.

[22] Hughes K, Borman AM (2018). Adiaspiromycosis in a wild European rabbit, and a review of the literature. J Vet Diagn Invest.

[23] Tobon JL, Yuill TM, Samuel WM (1976). Adiaspiromycosis in the Franklin’s ground squirrel, *Spermophilus franklini*, and pika, *Ochotona princeps*, from Alberta, Canada. J Wildl Dis.

