# Supplementary figures and images for: Adiaspore development and morphological characteristics in a mouse adiaspiromycosis model
Source: Vet Res. 2020 Sep 15;51:119. doi: 10.1186/s13567-020-00844-3 (PMC7493162; doi:10.1186/s13567-020-00844-3)

# Additional File 1

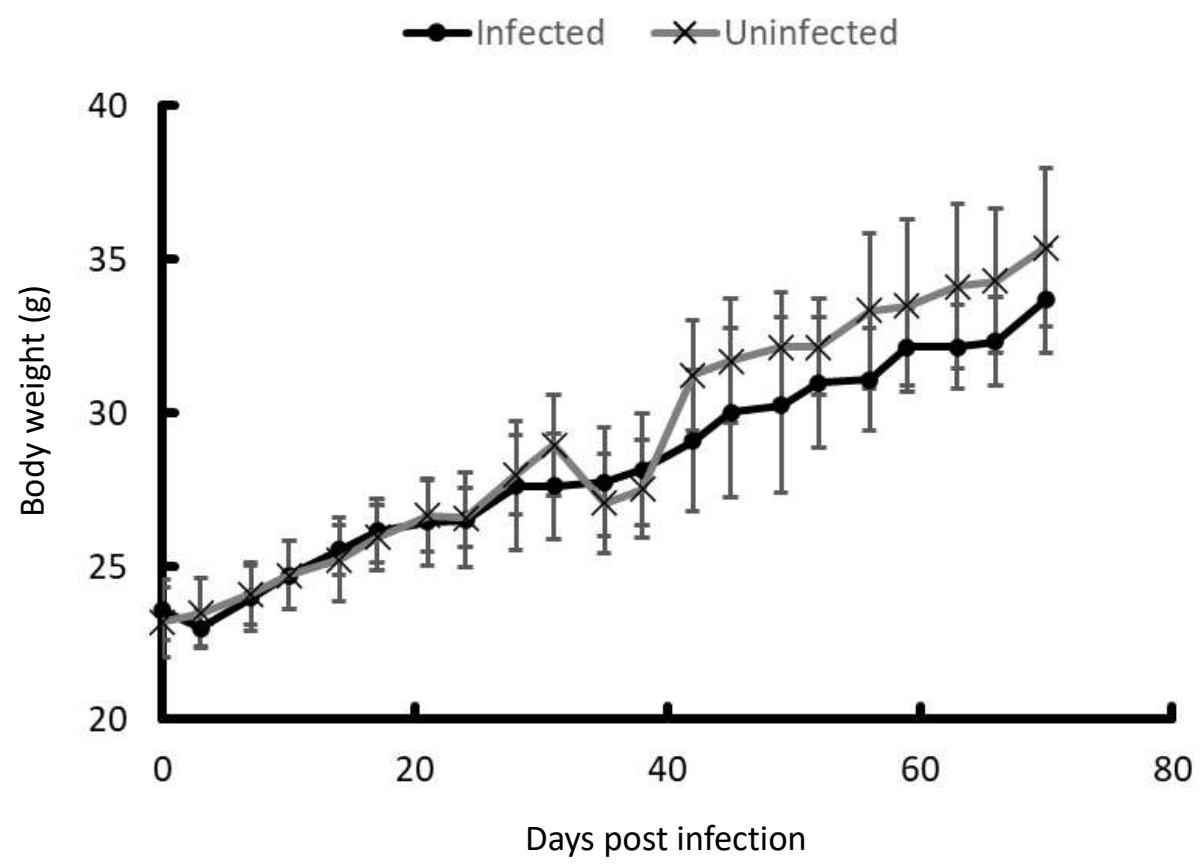

Supplement: Supplementary file 1 — Additional file 1. Body weights of mice infected with 4 × 10^6^ spores or uninfected. Error bars represent standard deviations of the means. [file 13567_2020_844_MOESM1_ESM.pdf]

## Additional File 2

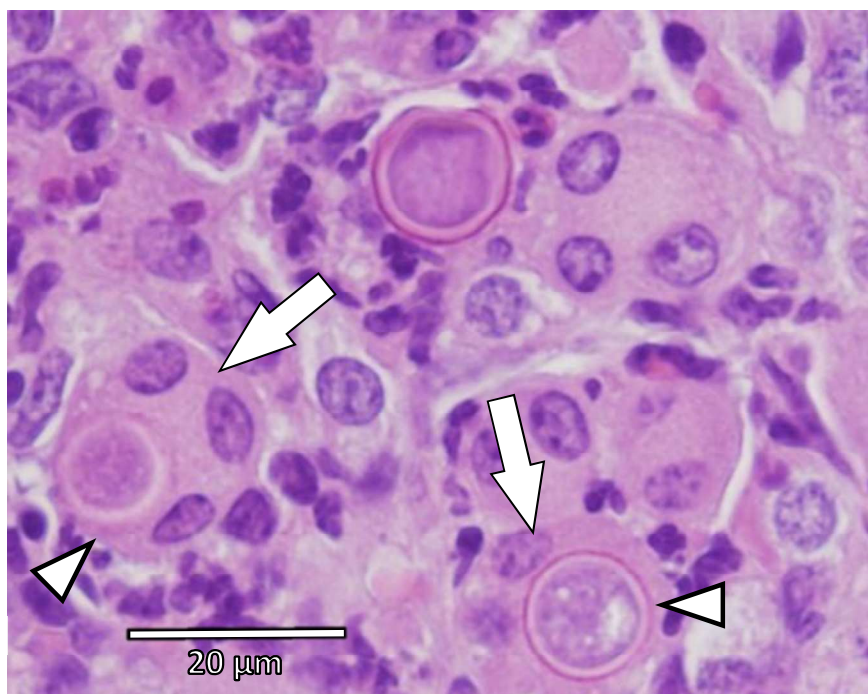

Supplement: Supplementary file 2 — Additional file 2. Adiaspores surrounded by multinucleate giant cells at 8 dpi. Arrows and arrowheads respectively indicate multinucleate giant cells and surrounded adiaspores. [file 13567_2020_844_MOESM2_ESM.pdf]

## Additional File 3

4 dpi

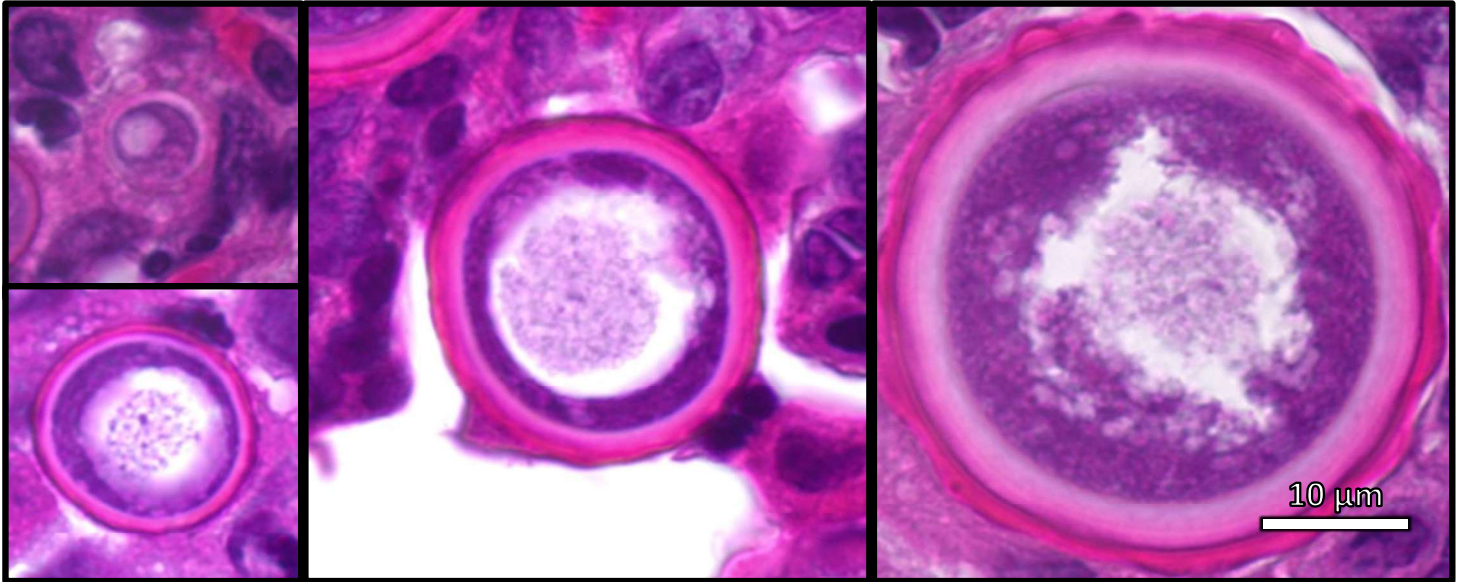

10 dpi

30 dpi

50 dpi

Supplement: Supplementary file 3 — Additional file 3. Adiaspores in mouse lungs at 4, 10, 30, and 50 dpi. [file 13567_2020_844_MOESM3_ESM.pdf]

## Additional File 4

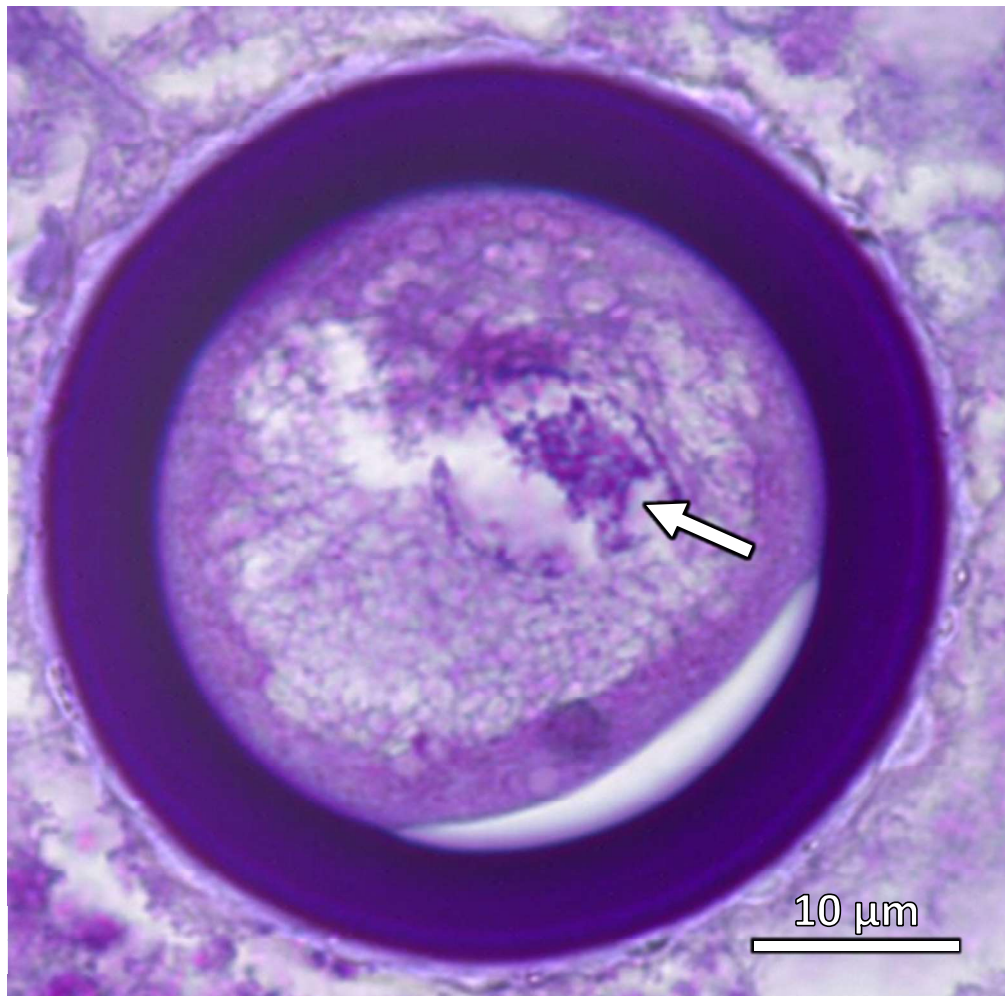

Supplement: Supplementary file 4 — Additional file 4. Representative adiaspores in which granular materials (arrow) were observed. The adiaspores are those of mouse lungs at 70 dpi, stained using PAS procedure. [file 13567_2020_844_MOESM4_ESM.pdf]

## Additional File 5

30 dpi

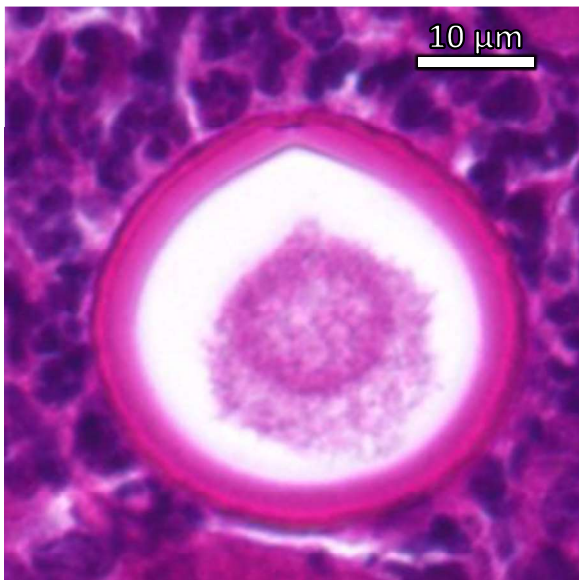

70 dpi

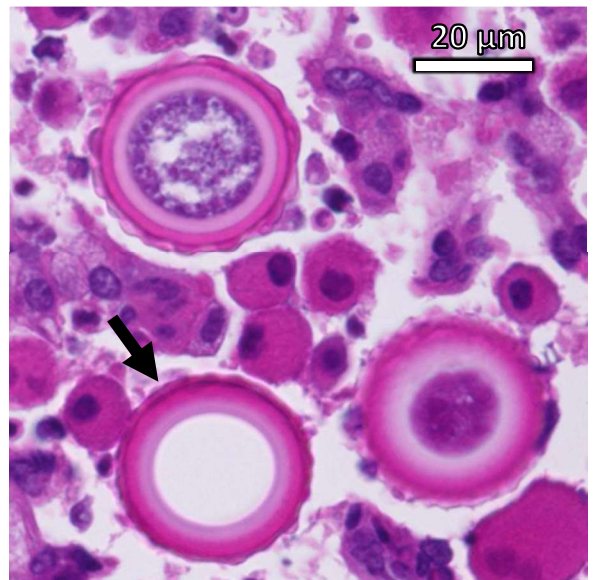

Supplement: Supplementary file 5 — Additional file 5. Adiaspores at 30 dpi (left) and 70 dpi (right) in mouse lungs. The adiaspore found at 30 dpi was not intact, but it retained debris-like materials. The adiaspore (arrow) found at 70 dpi was empty. [file 13567_2020_844_MOESM5_ESM.pdf]

## Additional file 6

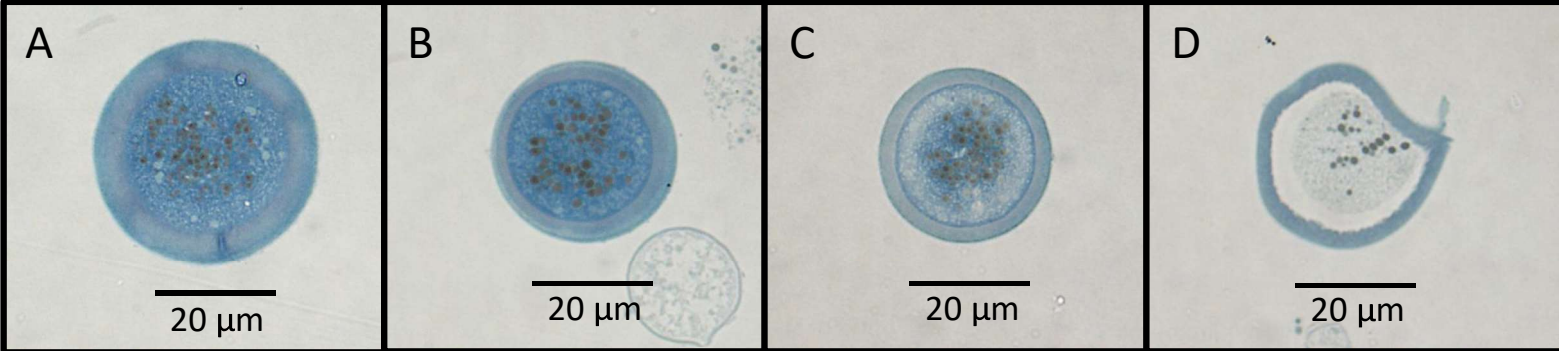

Supplement: Supplementary file 6 — Additional file 6. Adiaspore development in FBS in vitro. Adiaspores were stained with toluidine blue. [file 13567_2020_844_MOESM6_ESM.pdf]

# Additional file 7

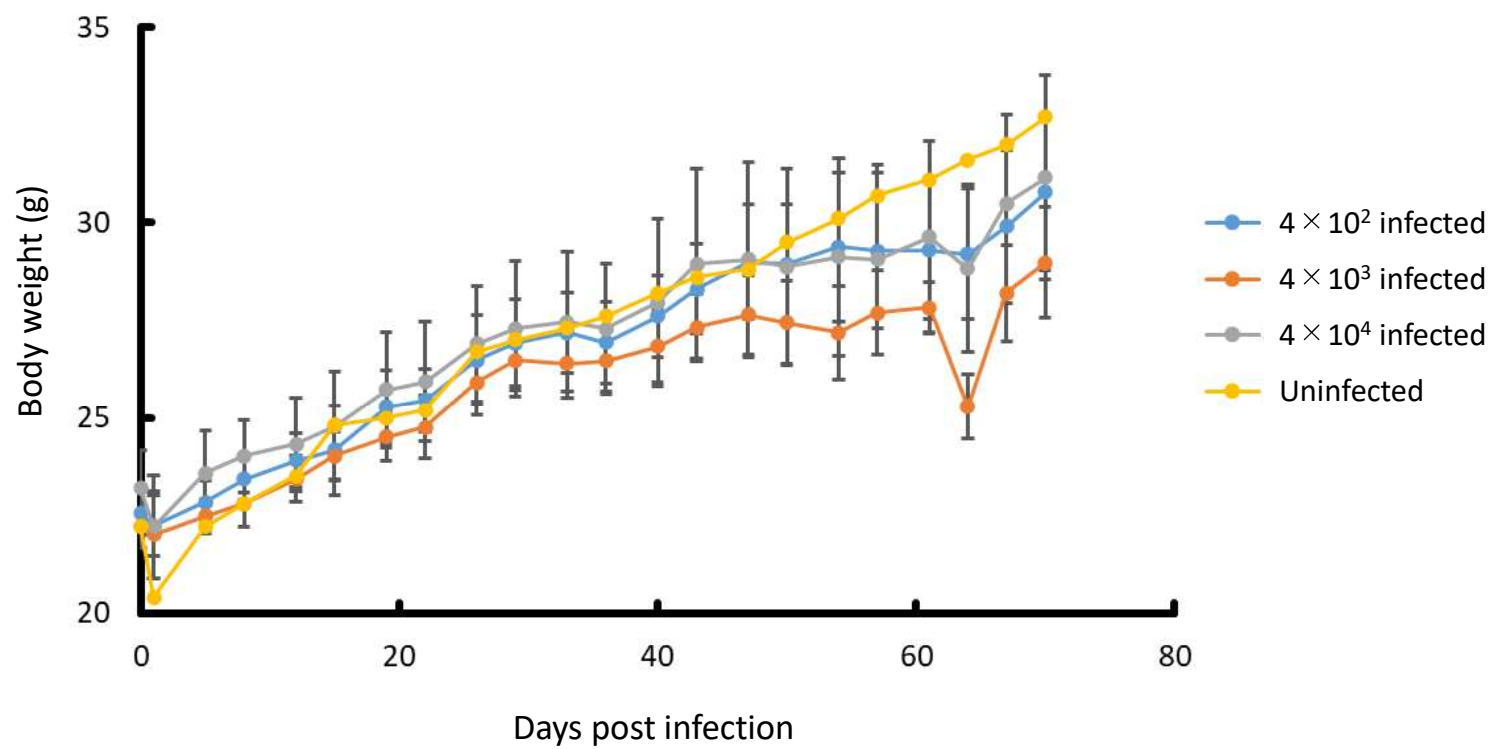

Supplement: Supplementary file 7 — Additional file 7. Body weights of mice infected with 4 × 10^2^, 4 × 10^3^, or 4 × 10^4^ spores, or uninfected. The error bars represent standard deviations of the means. [file 13567_2020_844_MOESM7_ESM.pdf]
